# Evaluation of Nudge-Based Notification for Follow-Up Examinations in Health Check-Ups Targeting Occupational Health Staff and Undiagnosed Workers: A Randomized Controlled Trial

**DOI:** 10.7759/cureus.70032

**Published:** 2024-09-23

**Authors:** Masaki Takebayashi, Yuri Mizota, Mira Namba, Yudai Kaneda, Kurenai Takebayashi, Hirohide Shibutani, Tatsuya Koyama

**Affiliations:** 1 Sociology, Aomori University, Aomori, JPN; 2 Public Health, Shizuoka Graduate University of Public Health, Shizuoka, JPN; 3 Medicine, Keio University, Tokyo, JPN; 4 Medicine, Hokkaido University, Sapporo, JPN; 5 Health and Wellness Planning, Olympus Corporation, Tokyo, JPN; 6 Human Life Sciences, Mimasaka University, Okayama, JPN

**Keywords:** follow-up examinations, health checkup, nudge, occupational health staff, workers

## Abstract

Purpose

Follow-up examinations after health check-ups are important for early detection of noncommunicable diseases among workers. Nudging can serve as an effective intervention for individuals who avoid follow-up examinations due to cognitive biases. This study aims to evaluate the interest in nudge-based notification for follow-up examinations, targeting occupational health staff and undiagnosed workers in a randomized controlled trial.

Methods

Participants were randomly assigned to either a control group (receiving a text-based notification without nudges) or a nudge group (receiving a notification that modified the control group based on Easy-type nudges). An anonymous web survey was administered.

Results

Occupational health staff (n = 425) rated all items significantly higher, including “willingness to use the notification” which scored 2.22 for the control group vs. 3.62 for the nudge group on a 1- to 5-point scale (P < 0.001). Among undiagnosed workers (n = 871), there was no significant difference in “willingness to apply for the follow-up examinations” (3.01 vs. 3.09; P = 0.272), but all other items were rated significantly higher in the nudge group (P < 0.05).

Conclusion

Based on these findings, we suggest that occupational health staff should use nudge-based notifications. However, increasing the willingness of undiagnosed workers to undergo follow-up examinations remains challenging. To achieve this goal, it is necessary to incorporate multiple nudge elements into notifications.

## Introduction

Noncommunicable diseases (NCDs) are a major global health concern, responsible for 41 million deaths annually and accounting for 74% of all deaths worldwide [1]. While NCDs are spreading rapidly, mainly in low- and middle-income countries, they remain a significant issue in high-income countries as well [1]. For example, in Japan, more than 1 million people die from NCDs each year, which is equivalent to 82% of all deaths [2]. NCDs have wide-ranging effects on labor market outcomes, such as employment status and income [2], a challenge made more serious by Japan's declining workforce due to low birth rates [3]. Considering that the incidence of NCDs is increasing among people from their 20s, early detection and timely intervention are vital for workers [1,4].

Workplace health check-ups play an important role in the early detection of NCDs among workers. Risk factors such as high blood pressure, overweight/obesity, hyperglycemia, and hyperlipidemia increase the likelihood of developing NCDs [1]. In Japan, employers are obligated to provide workers with periodic health check-ups conducted by doctors, and workers are obligated to undergo these check-ups, including those for elevated blood pressure, overweight/obesity, hyperglycemia, and hyperlipidemia [5]. However, follow-up examinations after health check-ups are not mandatory for workers with abnormal findings. In Japan, only 35.1%-46.2% of patients with hypertension, diabetes, and dyslipidemia undergo follow-up examinations [6].

Many individuals have cognitive biases and do not necessarily seek timely medical attention after receiving health information. For example, individuals with present bias, an inclination to choose an immediate, smaller reward over a delayed larger reward, tend to avoid cancer screenings [7]. Although prior research specifically addressing the cognitive biases that inhibit follow-up examinations is scarce, similar cognitive biases affecting cancer screening behavior may also be at play.

Nudging can be an effective intervention for individuals influenced by cognitive biases [8]. A nudge is defined as “any aspect of the choice architecture that alters people’s behavior in a predictable way without forbidding any options or significantly changing their economic incentives [8].” For example, individuals with a strong present bias, who tend to procrastinate when reading a promotion notification for health check-ups, are more likely to read it immediately if it is written with fewer words (a cognitive ease nudge) [9]. The Japanese government recommends the use of nudges to promote cancer screening, and these strategies could be applied to encourage follow-up examinations [10].

Although nudge-based notifications are recommended, some notifications for follow-up examinations have not yet incorporated nudges. Even if notifications designed with nudges are developed, they may not reach undiagnosed workers unless adopted by occupational health staff, such as company doctors or nurses [11]. Therefore, it is crucial for these notifications to be accepted by both occupational health staff and undiagnosed workers. To the best of our knowledge, no randomized controlled trials have examined the willingness of both groups to use nudge-based follow-up examination notifications.

This study aims to evaluate the willingness of occupational health staff and workers to engage in follow-up examinations based on the hypothesis that a nudge-based notification is more likely to be accepted by both groups than a conventional notification.

## Materials and methods

Research design

An intention-to-treat randomized controlled trial was conducted between two parallel groups in accordance with the Consolidated Standards of Reporting Trials (CONSORT) statement [10].

Targeting and assignment

This study had two targets: occupational health staff and workers with abnormal findings, such as hypertension, diabetes, and dyslipidemia, who had not undergone follow-up examinations in the previous year (hereafter referred to as undiagnosed workers).

Occupational Health Staff

Recruitment was conducted among participants of occupational health seminars in which the first author, Masaki Takebayashi, served as a lecturer and voluntarily agreed to participate. The exclusion criterion was age of < 20 years. The first author assigned them to two groups using a random number table. No financial incentive was provided for participating in the survey.

Undiagnosed Workers

Recruitment was conducted among individuals who voluntarily applied through an Internet survey company. The exclusion criteria were age < 20 years, occupational health staff, and part-time employment. Participants were randomly assigned to participate in a survey using the company’s system. Respondents were given an incentive worth up to USD 0.07 to complete the survey through the survey company.

Interventions and surveys

Two types of promotional notifications were developed as interventions for follow-up examinations. All interventions and surveys were conducted online between April and May 2024. Notifications for both groups were distributed online, and the participants were instructed to respond immediately upon receiving them.

Control Group

**Figure 1:**
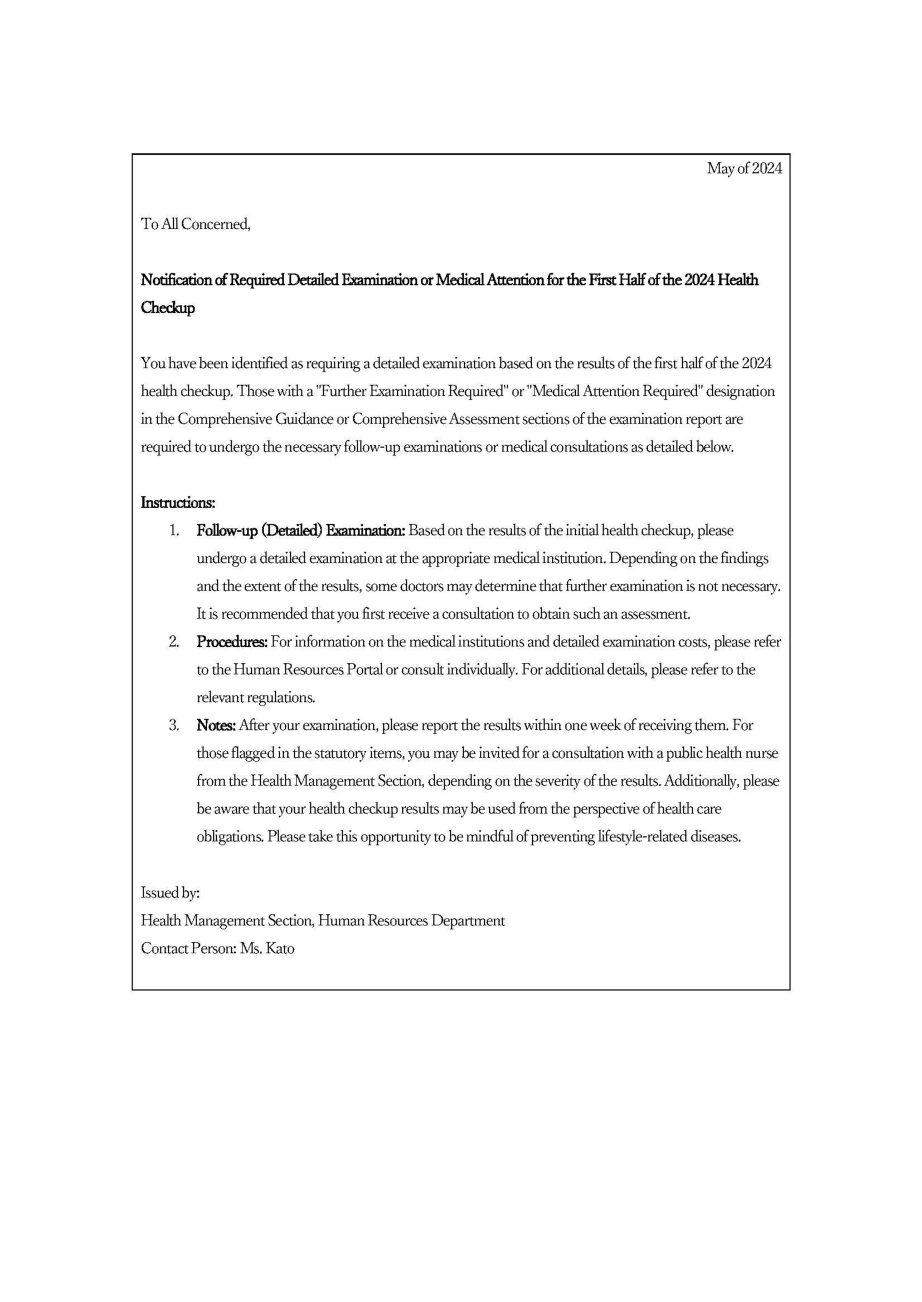
Promotion notification for follow-up examinations in the control group Image Credits: The first author.

A text-based notification, a slightly modified version of that used by the authors’ client companies, was used for the control group (Figure 1). External behavioral economists have confirmed that this notification did not contain nudge factors.

Nudge Group

We discussed the control group notification according to the nudge framework EAST (Easy, Attractive, Social, Timely) [12]. Several problems were identified such as “Information overload (lack of the Easy nudge),” “Difficult to find out which department to contact (lack of the Easy nudge),” “Too prominent risk expressions (counterproductive the Attractive nudge),” and “Bureaucratic impression by the all-black color scheme (lack of the Attractive nudge).” These were presumed to be particularly disliked by workers with strong present bias and risk aversion bias, which could inhibit conducting examinations [7].

**Figure 2:**
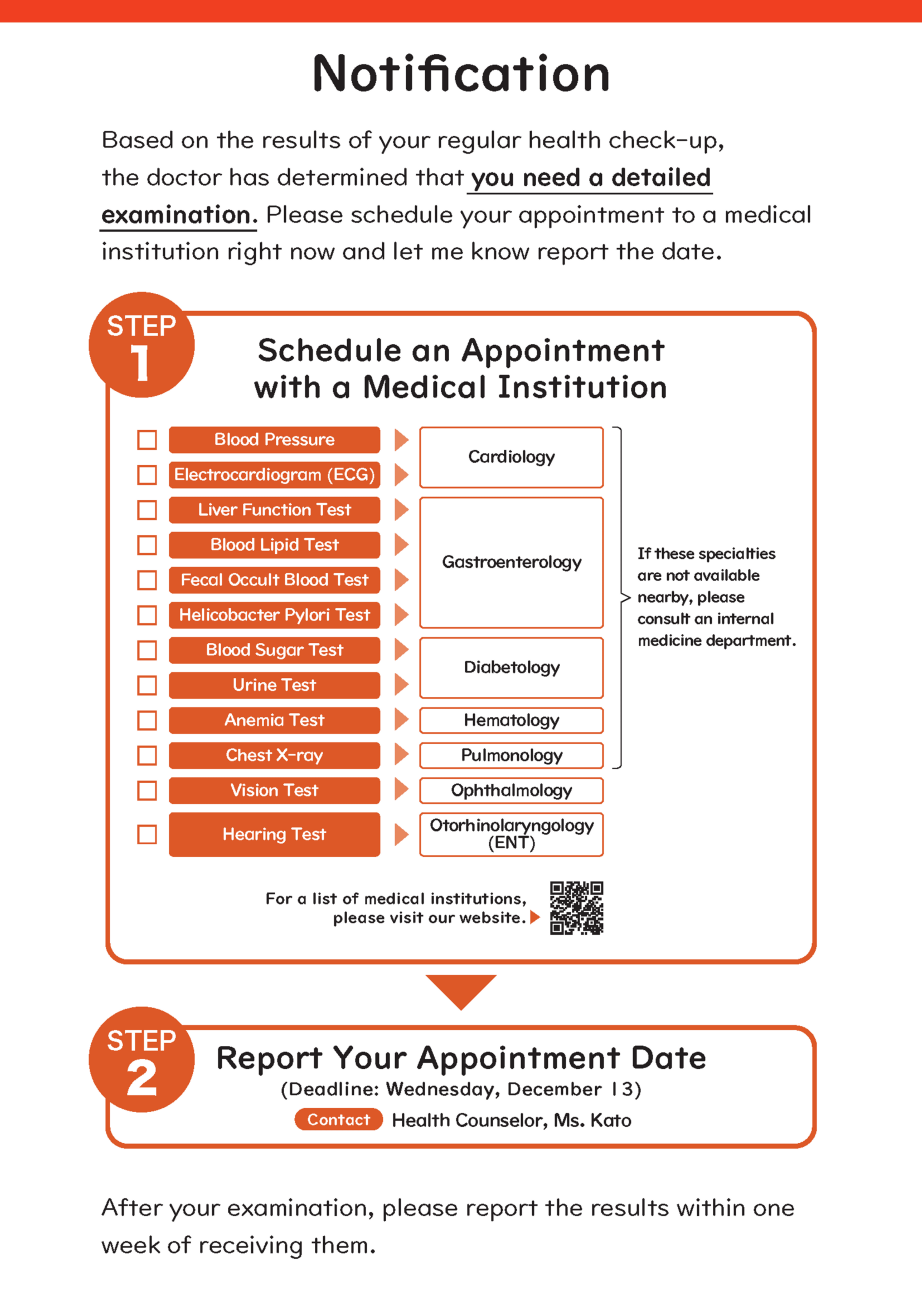
Promotion notification for follow-up examinations in the nudge group Image Credits: The first author. Detailed health risk information related to the check-up results was provided separately to those with findings in the follow-up examinations.

Based on these findings, the nudge group notification was developed mainly according to Easy Nudge (Figure 2). The text was simplified to reduce information overload by focusing on providing clear information on the relevant medical departments for each prevalent condition. The notice used orange and black schemes to attract attention. These nudges were designed based on the advice of external behavioral economists.

Outcomes

The primary outcomes were “willingness to use the notification and recommend it to other staff (occupational health staff) and “willingness to apply for follow-up examinations (undiagnosed workers).” The follow-up outcomes were “Easy to read” and “Easy to apply” (corresponding to the Easy nudge), “Attracting my attention” (corresponding to the Attractive nudge), “I felt many people would apply” (corresponding to the Social nudge), and “I wanted to read it immediately” (corresponding to the Timely nudge). The participants were also asked if they felt “uncomfortable.” All responses were given on a five-point scale from “Disagree (1)” to “Agree (5).” Additionally, a free-text comment section has been included to supplement the outcomes. The questions were designed in accordance with those used in previous studies [13]. An open-ended opinion box was also provided. Data on basic attributes such as sex and age were also collected. The first author grouped and categorized similar comments into “positive” and “negative” opinions, which were then reviewed by the other authors.

Ethical considerations

This study was approved by the Research Ethics Committee of the Aomori University (Approval No. 06-2023) and registered in the University Hospital Medical Information Network (UMIN) Clinical Trials Registry (UMIN000055068, registered on January 4, 2024). At the start of the study, participants were informed about voluntary participation and privacy compliance and that they would not face any disadvantages if they chose to discontinue the study. If they agreed, they were instructed to provide consent by checking a box. The surveys were conducted anonymously. After the study was completed, all the participants were provided with an opportunity to view both notifications.

Statistical analysis

We assumed a power of 80%, an α error of 5%, and an effect size of 20%. A minimum sample size of 412 was calculated using G*Power version 3.1.9.4 (Heinrich-Heine-Universität, Düsseldorf, Germany). Missing values were excluded from the analysis. Significance was examined using the t-test and chi-square test for basic attributes and the Mann-Whitney U test for outcomes. IBM SPSS Statistics 28 (IBM Japan, Tokyo, Japan) was used for the analysis, with a significance level of 5% (two-tailed test).

## Results

**Figure 3:**
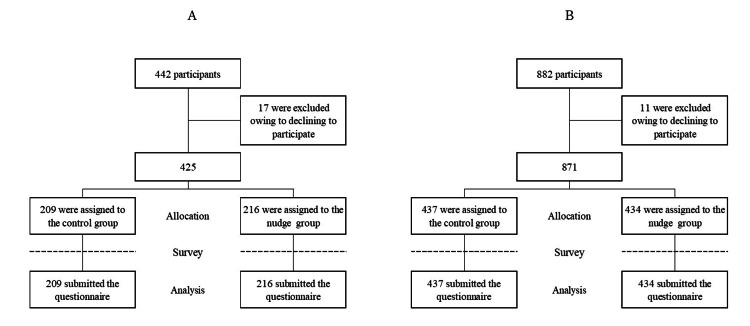
Trial profile (A) The flow diagram of occupational health staff, and (B) that of undiagnosed workers. We allocated the occupational health staff using a random number table, and the undiagnosed workers using the company’s system.

Of the 442 occupational health staff members who attended the online seminars, 425 responded (See Figure 3A). Additionally, of the 882 undiagnosed workers who signed up for the Internet survey, 871 responded (Figure 3B). The basic attributes of the participants are presented in Table 1. No significant differences were found across all the items.

**Table 1:** Basic characteristics of the participants A significance level was defined as P < 0.05 (two-tailed). Missing values were excluded from the analysis. ^a^ The comparison was made using chi-square tests. ^b^ The comparison was made using t-tests. ^c^ The comparison was made using a Mann-Whitney U test.

Occupational health staff						Undiagnosed workers					
Items	Control group (n=209)		Nudge group (n=216)		P value	Items	Control group (n=437)		Nudge group (n=434)		P value
	n	%, SD	n	%, SD			n	%, SD	n	%, SD	
Sex ^a^	-	-	-	-	0.786	Sex ^a^	-	-	-	-	0.879
Male	59	28.6%	58	27.0%	-	Male	262	60.0%	257	59.2%	-
Female	147	71.4%	157	73.0%	-	Female	175	40.0%	177	40.8%	-
Age ^b^	45.9	±10.8	46.4	±10.1	0.607	Age ^b^	47.9	±11.0	47.8	±11.3	0.833
Job ^a^	-	-	-	-	0.912	Smoking habit	-	-	-	-	0.517
Doctor/dentist	8	3.8%	7	3.2%	-	Smokers	113	25.9%	103	23.6%	-
Nurse	73	34.9%	80	37.0%	-	Non-smokers	324	74.1%	331	76.3%	-
Nutritionist	11	5.3%	13	6.0%	-	-	-	-	-	-	-
Clerical job	109	52.2%	105	48.6%	-	-	-	-	-	-	-
Others	8	3.8%	11	5.1%	-	-	-	-	-	-	-
Number of workers ^a^	-	-	-	-	0.955	Frequency of follow-up examinations in the past 10 years ^c ^	-	-	-	-	0.745
1-9	12	5.9%	14	6.7%	-	Always	37	8.5%	54	12.4%	-
10-99	61	29.8%	68	32.4%	-	Sometimes	171	39.1%	229	52.8%	-
100-999	76	37.1%	72	34.3%	-	None/others	231	52.9%	231	53.2%	-
1000-	50	24.4%	49	23.3%	-	-	-	-	-	-	-
Unclear	6	2.9%	7	3.3%	-	-	-	-	-	-	-

The results are summarized in Table 2. Among occupational health staff, the nudge group scored higher on all items than the control group. Undiagnosed workers also rated the nudge group significantly higher on all items except for “willingness to apply.”

**Table 2:** Degree of outcomes All responses were given on a five-point scale from “Disagree (1)” to “Agree (5).” A significance level was defined as P < 0.05 (two-tailed). Missing values were excluded from the analysis. The comparison was made using a Mann-Whitney U test. To ensure robustness, a Mann-Whitney U test was conducted, and the significance levels for all items remained unchanged.

Items	Occupational health staff					Undiagnosed workers					
	Control group (n=209)		Nudge group (n=216)		P value	Control group (n=437)		Nudge group (n=434)			P value
	Average	SD	Average	SD		Average	SD		Average	SD	
Willingness to use it	2.22	±0.89	3.62	±0.92	<0.001	-	-		-	-	-
Willingness to apply	-	-	-	-	-	3.01	±1.07		3.09	±1.04	0.272
Willingness to recommend it	2.18	±1.20	3.36	±1.21	<0.001	-	-		-	-	-
Easy to read	2.06	±1.02	3.71	±0.89	<0.001	2.73	±1.01		3.49	±1.03	<0.001
Easy to apply	2.04	±0.97	3.40	±0.97	<0.001	2.79	±0.95		3.20	±0.92	<0.001
Attracting attention	1.93	±0.84	3.34	±0.93	<0.001	2.73	±1.06		3.17	±0.92	<0.001
Feel that many workers would apply	2.26	±1.07	3.38	±0.94	<0.001	2.78	±0.97		2.91	±0.91	0.026
Want to read it immediately	2.11	±0.95	3.12	±0.97	<0.001	2.71	±1.00		2.91	±0.90	0.002
Unpleasant	3.20	±1.12	2.08	±0.89	<0.001	2.70	±0.97		2.50	±1.05	0.002

A total of 156 free-text comments were obtained: 34 from occupational health staff (24 from the control group and 10 from the nudge group) and 122 from undiagnosed workers (61 from the control group and 61 from the nudge group). The first author grouped and categorized similar comments into “positive” and “negative” opinions (See Table 3). Negative opinions were more prevalent among occupational health staff and undiagnosed workers, particularly in the control group.

**Table 3:** Free-text comments N/A: not applicable.

Occupational health staff		Undiagnosed workers	
Control group	Nudge group	Control group	Nudge group
(Positive: 0 opinions) N/A.	(Positive: 1 opinion) Easy to understand (n = 1).	(Positive: 9 opinions) Easy to understand (n = 6). Easy to report (n = 3).	(Positive: 29 opinions) Easy to understand (n = 16). Easy to report (n = 10). Easy to use (n = 3).
(Negative: 24 opinions) Difficult to read owing to excessive text (n = 14). Contains many difficult words (n = 5). Feels impersonal and cold (n = 3). Feels directed at a large audience owing to the lack of individual names (n = 2).	(Negative: 9 opinions) Unclear on what actions to take (n = 3). Reporting feels bothersome (n = 3). Contact information is hard to find (n = 2). Seems time-consuming and likely to be postponed (n = 1).	(Negative: 52 opinions) Difficult to read (n = 34). Lack of perceived necessity for follow-up examinations (n = 13). Feels bureaucratic (n = 5).	(Negative: 32 opinions) Unclear on what actions to take (n = 17). Lack of perceived necessity for follow-up examinations (n = 10). Feels imposing (n = 5).

## Discussion

Occupational health staff rated the nudge group significantly higher on all items, and the undiagnosed workers rated the nudge group significantly higher on all items, except for “Willingness to apply.” The hypothesis that nudge-based promotional notification is accepted by occupational health staff and undiagnosed workers was almost supported. However, it might not increase “Willingness to apply” for the undiagnosed workers. The background of the acceptance of many items and directions for improvement are summarized as follows.

Occupational health staff did not show a high willingness to use the notifications in the control group. The results suggest that existing notifications may be bottlenecks in the implementation process at the occupational health staff phase. The results showed significantly lower evaluations on all items; in particular, most negative opinions in the free-text comments were about appearance rather than content, which implies that the control group might not have been read positively. They seemed dissatisfied with the lack of cognitive ease, which tends to make people vigilant and suspicious [9]. This suggests that there is a bottleneck in the occupational health staff phase and that there is a potential need for improvement.

By contrast, the nudge group rated more than one point higher than the control group for all items. Occupational health staff had a high willingness to use the notification from the nudge group, and other items were also high, which meant that they were less likely to become bottlenecks at the occupational health staffing phase. Although the nudge group notification was designed with nudges aligned with the cognitive biases of undiagnosed workers, the occupational health staff responded positively. This finding suggests that occupational health staff may share the cognitive biases of the undiagnosed workers. Furthermore, in the nudge group, all items received higher ratings, indicating that the easy nudge design may have also promoted other nudge components.

Undiagnosed workers rated the nudge group notification significantly higher than the control group notification on all items except “Willingness to apply.” However, in the control group, undiagnosed workers scored lower than occupational health staff on all items, despite the notifications being designed to address undiagnosed workers’ cognitive biases. The reason for this finding remains unclear.

As for the nudge group, the positive results on “Easy to read,” “Easy to apply,” and “Want to read it immediately” show that the Easy and Timely nudge might work. Some people with health risks such as diabetes tend to have a strong present bias and procrastinate follow-up examinations, even with minimal friction costs [12,14,15]. These results imply that the friction cost of a follow-up examination may be due to poor visibility and the difficulty in choosing a department. Procrastination can be mitigated through simple instructions on behavior. This is consistent with previous reports, such as “Reduce the hassle factor” and “Simplify messages” [12,14,15].

A significant impact on “Attracting attention” in the nudge group suggests that the notification with two colors could have a function of the Attractive nudge. This simple coloring would be useful because excessive flamboyance could detract from the easy-nudge elements.

The positive impact of “Feels that many workers would apply” suggests the Social nudge might work. Some undiagnosed workers are smokers and obese, with strong peer bias [16-19]. Therefore, they will be more inclined to take the follow-up examinations if they feel that “Others may also do so.” This notification did not include the message “Many workers are also taking the follow-up examinations,” but it could create an atmosphere that evokes a peer bias.

“Unpleasant” was significantly reduced in the nudge group. Those who do not undergo health check-ups may fear being blamed for their unhealthy habits or having to change their lifestyles [20-22]. The results may indicate how disgusted they were with the existing notifications. Negative emotions, such as disgust, can lead to an underestimation of health risks, which may inhibit application [23]. The notification in the nudge group could help reduce this emotion of disgust.

However, the main outcome, “Willingness to apply,” did not increase significantly among the undiagnosed workers in the nudge group. Some reasons could be suggested from the free-text opinions, such as “unclear on what actions to take,” “lack of perceived necessity for follow-up examinations,” and “feels imposing.” This implies a need to improve the notification of the nudge group. Considering that the most effective interventions combine different elements, notifications may be better at incorporating multiple nudge elements [24]. Incorporating all elements of the EAST may compromise easy elements because of information overload [25]. Therefore, it is necessary to investigate appropriate combinations of nudges further.

This study has some limitations. First, the participants were recruited online, which may have attracted individuals with a higher motivation for health, potentially introducing selection bias. Second, some bias might have occurred among undiagnosed workers due to the USD 0.07 incentive. However, we assumed that there would be no major problems, as participants were randomly assigned to each group and the incentive was small. Third, the number of occupational health staff included in the analysis was smaller than the required sample. Fourth, this study aimed to understand the willingness of the two groups; however, it could not assert whether occupational health staff actually adopted nudge-based notifications, or whether workers actually underwent follow-up examinations. Further research is required to overcome these limitations.

On the other hand, this study has several strengths. First, it was conducted as a randomized controlled trial with both a control group and a nudge group. A systematic review reported that many studies on nudges in public health lifestyle interventions lack a control group, suggesting that such studies might be rare [26]. Second, this study was implemented in Japan, a country known internationally as a “cautiously pro-nudge nation [27].” Initially, there was a concern that the nudge group might experience unpleasant feelings because of misunderstandings about nudges [28]. Under such circumstances, the result that unpleasant feeling was reduced by using nudges might serve as a reference for promoting follow-up examinations in other countries. Finally, the questionnaires were conducted for the primary clientele, occupational health staff, and the secondary clientele, the undiagnosed workers. The findings of this study showed that the nudge-based notification did not create bottlenecks in the occupational health phase. This study is practical, and there are plans to implement the findings in companies.

## Conclusions

A randomized controlled trial (control group vs. nudge group) was conducted to evaluate the willingness to receive nudge-based follow-up examinations for occupational health staff and undiagnosed workers. Occupational health staff rated all items significantly higher, including “willingness to use the notification.” Among undiagnosed workers, there was no significant difference in “willingness to apply for the follow-up examinations”; however, all other items were rated significantly higher in the nudge group. These findings suggest that occupational health staff might use nudge-based notifications. However, increasing the willingness of undiagnosed workers to undergo follow-up examinations remains challenging. To achieve this goal, it is necessary to incorporate multiple nudge elements into notifications.
